# Supplementary material for: Inflammatory factors and risk of lung adenocarcinoma: a Mendelian randomization study mediated by blood metabolites
Source: Front Endocrinol (Lausanne). 2024 Aug 27;15:1446863. doi: 10.3389/fendo.2024.1446863 (PMC11384989; doi:10.3389/fendo.2024.1446863)
Supplement: Supplementary file 1 [file DataSheet1.zip › supplementary files/S2/GCST90200866/sensitivity-analysis.pdf]

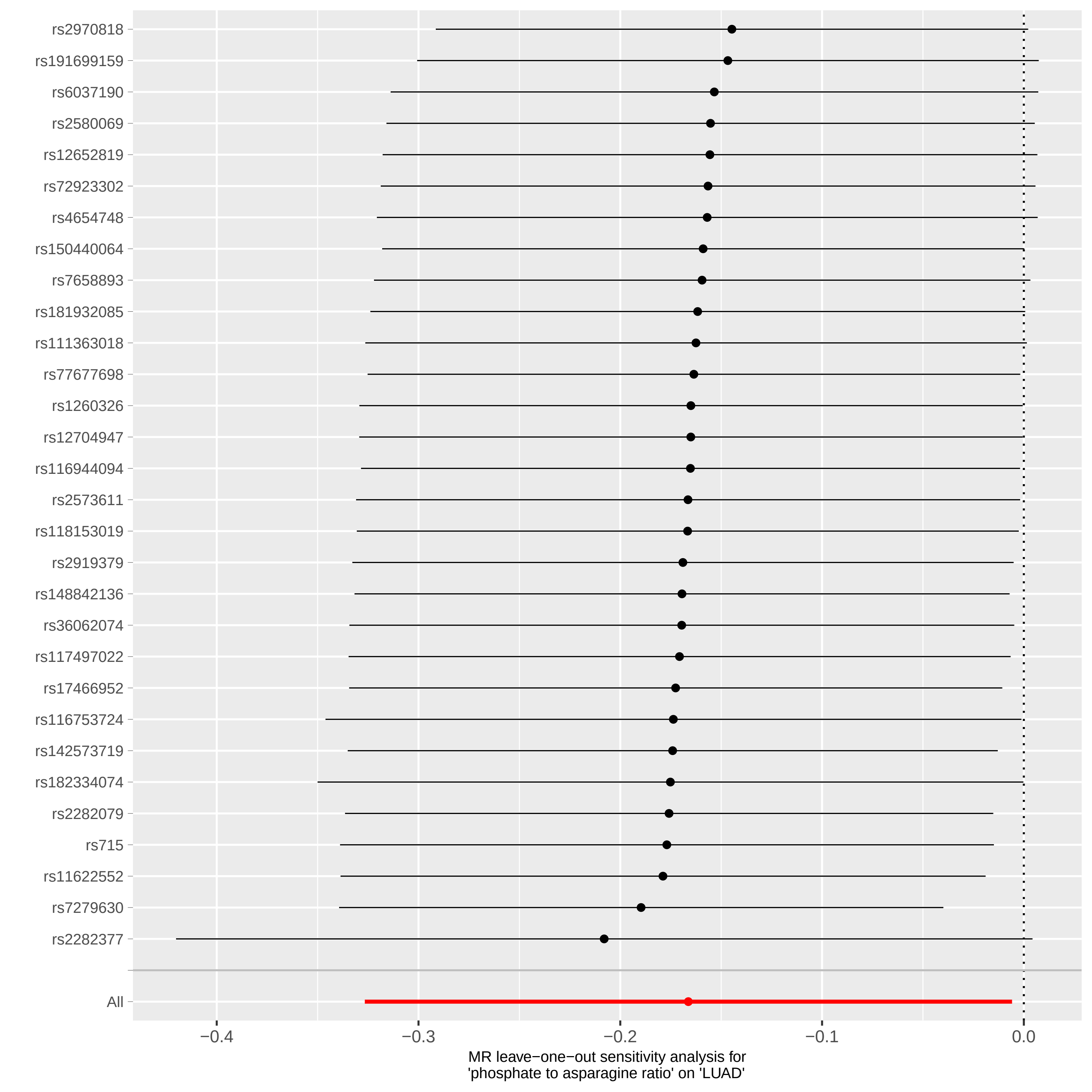
rs2970818
rs191699159
rs6037190
rs2580069
rs12652819
rs72923302
rs4654748
rs150440064
rs7658893
rs181932085
rs111363018
rs77677698
rs1260326
rs12704947
rs116944094
rs2573611
rs118153019
rs2919379
rs148842136
rs36062074
rs117497022
rs17466952
rs116753724
rs142573719
rs182334074
rs2282079
rs715
rs11622552
rs7279630
rs2282377
All
−0.4
−0.3
−0.2
−0.1
0.0
MR leave−one−out sensitivity analysis for
'phosphate to asparagine ratio' on 'LUAD'
